# MicroRNAs: As Critical Regulators of Tumor- Associated Macrophages

**DOI:** 10.3390/ijms21197117

**Published:** 2020-09-27

**Authors:** Bilash Chatterjee, Priyanka Saha, Subhankar Bose, Devendra Shukla, Nabanita Chatterjee, Sanjay Kumar, Prem Prakash Tripathi, Amit Kumar Srivastava

**Affiliations:** 1 Cancer Biology & Inflammatory Disorder Division, CSIR-Indian Institute of Chemical Biology, Kolkata, WB 700032, India; luke.dc2@gmail.com (B.C.); priaz12@rediff.com (P.S.); sbose33186@gmail.com (S.B.); devs2092@gmail.com (D.S.); 2 Chittaranjan National Cancer Institute, 37, S. P. Mukherjee Road, Kolkata, WB 700026, India; nabanita.chatterje@yahoo.com; 3 Division of Biology, Indian Institute of Science Education & Research, Tirupati, Andhra Pradesh 517507, India; sanjay28@gmail.com; 4 Cell Biology & Physiology, CSIR-Indian Institute of Chemical Biology, Kolkata, WB 700032, India; prem.tripathi@iicb.res.in

**Keywords:** tumor microenvironment (TME), microRNA (miRNA), dendritic cells (DCs), interleukins (ILs), tumor-associated macrophages (TAMs), tumor-necrosis factor-α (TNF-α)

## Abstract

Emerging shreds of evidence suggest that tumor-associated macrophages (TAMs) modulate various hallmarks of cancer during tumor progression. Tumor microenvironment (TME) prime TAMs to execute important roles in cancer development and progression, including angiogenesis, matrix metalloproteinases (MMPs) secretion, and extracellular matrix (ECM) disruption. MicroRNAs (miRNAs) are critical epigenetic regulators, which modulate various functions in diverse types of cells, including macrophages associated with TME. In this review article, we provide an update on miRNAs regulating differentiation, maturation, activation, polarization, and recruitment of macrophages in the TME. Furthermore, extracellular miRNAs are secreted from cancerous cells, which control macrophages phenotypic plasticity to support tumor growth. In return, TAMs also secrete various miRNAs that regulate tumor growth. Herein, we also describe the recent updates on the molecular connection between tumor cells and macrophages. A better understanding of the interaction between miRNAs and TAMs will provide new pharmacological targets to combat cancer.

## 1. Introduction

TME contains fibroblast cells, stromal cells, epithelial cells, adipocytes, B-cells, T-cells, mast cells, pericytes, macrophages, et cetera. Among these cells, tumor infiltrating macrophages play an indispensable role in tumor growth and progression [1]. The origin of macrophages is from monocytes. Under the inflammatory condition, circulating monocytes exit to the peripheral blood and further differentiate into a subset of tissue macrophages and dendritic cells (DCs) [2]. Macrophages exhibit remarkable heterogeneity in terms of phenotype and function in different tissue environments. However, macrophages residing in tissue are originated from yolk-sac-derived erythroid-myeloid progenitors [3,4]. Over the last two decades, accumulated data suggest that TME contains a significant population of TAMs, which play an imperative role in cancer development such as lung and ovarian [5,6]. The published reports suggest a positive correlation between the TAM population and poor prognosis of various malignancies, counting breast, prostate, and bladder cancers [7,8,9,10].TAMs play a key role in the cancer development by increasing cancer cell mobility, activation of MMPs, angiogenesis, extravasation, and immunosuppressive activity [6,11]. Another vital characteristic of macrophages is phenotypic plasticity. Based on the cellular and molecular stimuli and function, macrophages may be broadly categorized into classically-activated macrophages (also known as M1-like macrophages: pro-inflammatory) and alternatively-activated macrophages (M2-like macrophages: anti-inflammatory) [12]. In the presence of various stimuli, such as interferon (IFN)-γ, lipopolysaccharide (LPS) and other microbial infections, M1-like macrophages are induced, which are specified by generation of TNF-α, nitric oxide or other reactive oxygen species (ROS), and interleukins (ILs), such as IL-12, IL-23, IL-6, IL-1, and IL-18. Thus, the M1-like phenotype exhibits a high potential to kill cancer cells and microorganisms. Whereas key activating stimuli, such as glucocorticoids, IL-13, IL-10, IL-4, TLR ligands and immune-complexes induce the maturation of monocytes into the M2-like macrophages, which display Th2 response, matrix deposition, tissue repair and enhanced tumor progression and metastasis [13,14]. Besides, M1 and M2-like phenotypes, macrophages exhibit a broad spectrum of intermediate phenotypes between these two ends [13,14].

MiRNAs are tiny, single-stranded, 19–24 nucleotides long, endogenous, non-coding RNAs (nc RNAs). Substantial research data depict that miRNAs are capable of turning-off the gene expression either by degrading messenger RNA (mRNA) or translational repression. Within the nucleus, RNA polymerase II transcribes the miRNAs encoding genes into primary miRNAs, which are further processed by a series of enzymes, such as RNases III endonuclease, Drosha and Dicer, to form mature miRNAs [15]. Multiple studies have demonstrated that TAMs have aberrant expression of miRNAs [16,17,18]. MiRNAs can regulate tumor initiation, progression, and metastasis via targeting tumor promoter or suppressor genes. Based on their effects on tumor growth and progress, miRNAs can be divided into oncogenic or tumor-suppressive [19,20,21,22]. In addition, miRNAs exert their impacts in a tissue-specific manner. For instance, a particular miRNA can function as either oncogenic or tumor-suppressive depending on the cancer type [23,24].

Moreover, emerging preclinical and clinical reports depict that aberrant expression of few miRNAs may act as prognostic and diagnostic biomarkers in a broad array of human malignancies [25,26,27]. In recent times, a series of experimental studies have deciphered the importance of miRNAs in several immune cells, together with monocytes and macrophages. Over the last few decades, evidences accumulated from studies have shown the significance of macrophages and their precursors cells in developing tumor mass. Cross-talk between tumor-infiltrating macrophages and tumor cells is vital for the development of heterogeneous populations in tumor mass, as well as in progression and metastasis. MiRNAs are known to regulate heterogeneous tumor development at their various stages.

Herein, we spotlight mechanistic insights of the regulation of endogenous miRNAs in the maturation, differentiation, pro-tumoral, anti-tumoral, and immunosuppressive functions of macrophages. Understanding the crosstalk among miRNAs and immune cells, especially TAMs will uncover the new drug targets.

## 2. Diverse Strategies Opted by TAMs to Promote Tumor Progression—A Sneak Peek into Molecular Mechanisms

TAMs represent a significant population of immune cells in primary tumors and play an essential role in cancer development [7,8,9,10]. Based on the clues received from TME, TAMs exhibit remarkable phenotypic and functional heterogeneity. They can acquire either M1 or M2-like phenotype, as well as various intermediate phenotypes between two ends [13,14]. It has been suggested that TAMs residing in TME mostly display M2-like phenotype [5,7]. Over the last two decades, through extensive experimental and clinical research, it has been shown that tumor cells and various types of immune cells co-inhabit in almost all stages of neoplastic development [28]. One of the most prominent groups among these immune cells is the TAMs. Several lines of evidences suggest that TAMs act as pro-tumoral and exert their immunosuppressive role in TME by secreting certain growth factors, chemokines and cytokines and, suppressing T-cell activation. In doing so, TAMs have pronounced effects in enhancing chemoresistance, angiogenesis, tumor cell proliferation, metastasis, EMT, and immune evasion. Emerging studies suggest that TAMs might be used as predictive biomarkers for diagnosis of many types of cancers and possible pharmacological targets in cancer therapy [29].

### 2.1. Proliferation and Growth of Cancer Cells

Since macrophages have different subtypes, they may play different roles in cancer development. It has been found that M1-like macrophages can play a significant role in the initial stages of cancer progression while M2-like macrophages generally participate in later stages [30,31]. Given that TAMs secrete various growth promoting factors, such as EGF, FGF, et cetera, in the TME, which causes proliferation of cancer cells. Moreover, it was observed that lactic acid produced by cancer cells promotes tumor growth via enriching M2-like macrophages as well as enhancing vascular endothelial growth factor (VEGF) expression [32]. TAMs increase the ovarian cancer tumor growth via secreting developmental transcription factors including GATA binding protein-3 (GATA-3) through exosomes [33].

### 2.2. Angiogenesis

Macrophages are well known to facilitate the angiogenesis process, which leads to a poor prognosis in many cancers. The precursor cell linage of macrophages may move to tumor sites where they can differentiate into macrophages. TAMs secrete various soluble angiogenesis promoting mediators such as MMPs, basic fibroblast growth factor (bFGF), cathepsins, heparinase, uPA, platelet-derived growth factor-F (PDG-F), macrophage-inhibitory factor, VEGF, chemokines and cytokines like C-C motif chemokine ligand 2 (CCL2), CXCL8, IL1-β, transforming growth factors (TGF)-α and -β [34,35]. A positive correlation between the angiogenesis process and an abundance of TAMs has been noticed in breast carcinoma. Under hypoxia, activation of HIF-2α increases the recruitment of macrophages, which could promote the angiogenesis process [36]. It is suggested that HIF-2α can also induce VEGF expression, a key player of angiogenesis [36,37]. Emerging evidence suggests that CCL2 is important for the recruitment of TAMs in TME [38]. Since TAMs can secrete CCL2, which in turn recruit more macrophages at the tumor site, it may play an indispensable role in the modulation of angiogenesis and cancer progression [39]. In addition, TAMs secrete CCL18 that facilitates angiogenesis and tumor growth in breast carcinoma [40].

### 2.3. Metastasis

TAM regulates almost every step of cancer progression through the production of a series of signaling molecules such as cytokines, growth factors, chemokines, and inflammatory molecules. Cytokines like TGF-β and CCL18 secreted by TAMs can induce invasive phenotype and mesenchymal markers in tumor cells [28]. TNF-*α* released by TAMs has been reported to upregulate the transcription factors such as Snail-1 and Zeb-1/2, which results in enhanced tumor growth via downregulating E-cadherin expression [41]. The tumor-derived colony-stimulating factor-1 (CSF-1)/EGF paracrine loop helps in the recruitment of TAMs around the blood vessels, which promotes cancer cell escape through various mechanisms. TAMs derived proteolytic enzymes cathepsins B or S, (a serine protease) and MMPs degrade the ECM components, thus facilitating the generation of the pre-metastatic niche [28,42].

Interestingly, Dhanasekaran et al. demonstrated that MYC and Twist-related protein 1 (TWIST1) regulate transcriptional program in cancer cells, which in turn induces a plethora of cytokine production such as IL-13, CCL2, CCL7, CCL5, and CXCL1 that mediates the TAMs recruitment and polarization [43]. Guo et al. have also observed that EGF, secreted from TAMs, activates the EGF-EGFR pathway that contributes to EMT progression [31]. Apart from that, TAMs release chemokines like CCL18, which can induce EMT in endothelial cells [40]. Furthermore, in pancreatic cancer cells, TAMs can induce EMT via modulating TLR4/IL-10 axis [44].

### 2.4. Resistance to Chemotherapy

The role of TAMs in imparting chemoresistance has been reviewed by Chen et al. [45]. In progressive solid tumors, the resistance to chemotherapy and radiotherapy is widespread. Accumulated experimental data have confirmed the involvement of TAMs in chemoresistance and hence, targeting TAM population could be useful in the suppression of therapy resistance and tumor relapse [45]. As demonstrated by Paulus et al., the CSF-1 kinase receptor targeting antibody significantly sensitizes breast cancer cells to the chemotherapeutic drug [46]. TAM secretes IL-6 with pleiotropic potential, which has been reported to contribute chemoresistance in breast cancer [45,47]. Furthermore, milk fat globule epidermal growth factor VIII (MFG-E8) in TAMs enhances cisplatin resistance in tumor cells in concert with IL-6 via regulating the signal transducer and activator of transcription-3 (STAT-3) and hedgehog (Hh) signaling [45,48]. Moreover, IL-10 and IL-34 were found to mediate chemoresistance in different malignancies [45,48].

### 2.5. Immunosuppression

TAMs could exert their immunosuppressive functions in TME through various mechanisms [28]. It is suggested that TAMs can directly impair T-cell functions via multiple mechanisms, such as inhibiting the proliferation of naïve T-cell, degradation of metabolites needed for T-cell division, and suppressing T-cell activation via producing certain cytokines and chemokines [49,50]. It is evident that TAMs may hamper the tumor-killing ability of tumor-infiltrating NK and T-cells thus creating an immunosuppressive niche in TME with other immune cells [51,52]. Various cytokines and chemokines secreted by M2-like phenotypes such as CCL5, CCL18, CCL17, CCL22, CCL20, CCL24, VEGF, PDGF-B, IL-10, TGF-β, and prostaglandin eotaxin 2 (PGE2) are established immune suppressors [34,45]. TGF-β, PGE2, and other chemokines from TAMs impair the DCs maturation, which destroys the balance between innate and adaptive immunity [34,53]. Programmed death-ligand 1 (PD-L-1) expressed by TAMs, the ligand for PD-1 in T-cells, is an active mediator of immunosuppression [31,54]. In addition, TAMs may produce enzymes such as arginase-1 (ARG-1) and nitric-oxide synthase (NOS) which can inhibit T-cell function [55,56].

## 3. MiRNAs Involved in Regulation of Macrophage Differentiation and Maturation

Macrophages are mostly derived from hematopoietic stem cells (HSCs), an embryonic progenitor, via a multiple-step process. HSCs differentiate into myeloid progenitor (LMP), which under-regulated conditions, further differentiate into granulocyte–monocyte progenitor (GMP), and then form monocytes. Monocytes are precursors of macrophages. They enter in blood vessels and reach different organs such as the liver, brain, heart, et cetera, to develop tissue-specific macrophages. Almost all types of tissue-resident macrophages pose some sort of functional similarities in regard to their phagocytic activity. However, based on the site of maturation and growth, macrophages exhibit diverse phenotypes and functions. For example, lung alveolar macrophages, bone osteoclasts, liver Kupffer cells, brain microglia, or peritoneal macrophages are evolved to execute tissue and organ specific functions [57,58]. Substantial evidence has suggested the importance of miRNAs as the primary driver of HSC self-renewal or differentiation. Additionally, it was observed that TAMs are unable to grow and survive in TME for a long duration [11]. Therefore, it is imperative to continuously recruit precursor cell lineage in TME to renew and maintain the macrophage population. A panel of miRNAs is involved in HSC differentiation and maturation. Roy has reviewed the miRNAs implicated in HSCs maturation in the human and mouse model [59]. Arsenic resistance protein 2 (Ars2) is an RNA binding protein that is involved in processing and maturation of miRNAs, Ars2 depleted mouse developed impaired bone marrow thereby miRNA plays an indispensable role in HSC fate determination [60]. Connell and co-workers observed that the maintenance of HSCs in mouse bone marrow was accompanied by higher intracellular expression of miR-29a, miR-125a-5p, miR-125b-5p, miR-126-3p, miR-130a, and miR-155 [61]. However, reduced levels of miR-130a and miR-126 triggered the differentiation of HSCs in mature progeny [62]. Guo et al. have shown that miR-125a can regulate the stem cell pool via regulating BAK1 mediated-HSC apoptosis [63]. Downregulation of miR-126 using lentiviral-based sponges significantly enriches the HSC pool via targeting the PI3K/AKT/GSK3β axis. Thus, miR-126 is vital for the maintenance and activation of HSC, reflecting the significance of miRNAs in the maturation of HSC [64]. PU.1 is a transcription factor expressed in early T-lymphoid, B-lymphoid, monocytic and granulocytic cells [65]. PU.1 regulates the development of myeloid lineages from HSCs progenitors and impact the differentiation of HSC into LMP through regulating the levels of miR-155, miR-146a, miR-338, and miR-342 [66]. Overexpression of miR-146a promotes differentiation of HSCs while its inhibition hampers the development of macrophages in early zebrafish development [66]. Furthermore, PU.1 inhibits the expression of the oncogenic miR-17-92 cluster, which transcribes seven miRNAs involved in regulation of the various key cellular processes. PU.1 curtails the miR-17-92 cluster expression via targeting Egr2. Egr2 promotes histone demethylation in CpG island located at miR-17-92 promoter via recruiting histone demethylase Jarid1b [67]. The CCAAT/enhancer-binding protein (C/EBP) is necessary for the conversion of LMP to GMP. It increases the human-miR-223 expression, which promotes LMP differentiation [68]. The enhanced level of PU.1 facilitates the differentiation and maturation of GMP to macrophages. In both humans and mice, miR-21 and miR-196b facilitate the differentiation of GMP to monocytes [69]. Moreover, macrophages exhibit elevated miR-424-5p, miR-362-3p, miR-335-5p, and miR-106-3p compared to progenitor cells, which depicts that these miRNAs could promote maturation of macrophages [70]. The detailed list of miRNAs regulating macrophage differentiation and maturation has been shown in Table 1. Overall, these studies suggest that the cooperative action of multiple miRNAs regulates the macrophage development and maturation process in both humans and mice. Unraveling the mechanism of miRNA-mediated monocyte differentiation will likely provide candidate targets for pathophysiology.

## 4. MiRNAs Involved in Macrophage Activation and Polarization

**Figure 1:**
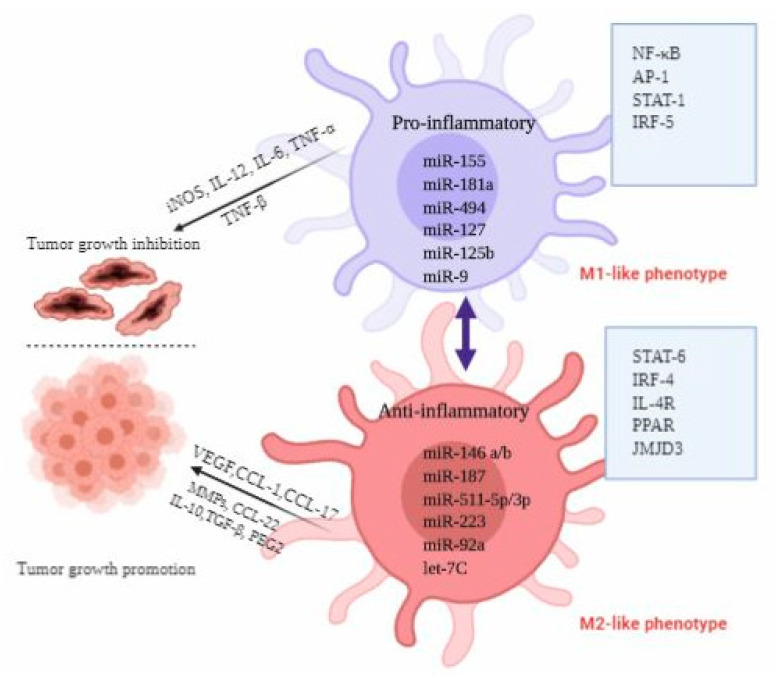
miRNAs regulating macrophage activation and polarization.

Based on the molecular signals received from TME, macrophages exhibit a continuum of phenotypes to perform different cellular and biochemical functions. We can have a general classification of macrophages by grouping them into M1 and M2-like phenotypes. Furthermore, on the basis of activation status and functional heterogeneity, M2-like macrophages may be categorized into four subsets: M2a, M2b, M2c, and M2d [42]. The detailed description of these subsets has been given in Table 2. TAMs constitute the major portion as inflammatory cells in TME; they display the similar morphological and functional characteristics as M2-like macrophages [5,7,113]. Therefore, TAMs are recognized as new subgroup of M2d [42]. Of note, the clear classification of macrophages subtypes is complex. It is difficult to identify a specific type of macrophages using single set of specific markers. One main feature of all M2-like macrophages is the generation of ARG-1 enzyme that reduces T-cell activation [55,114]. Graff and co-workers performed the miRNA profiling studies on mammalian macrophages to identify the miRNAs associated with macrophage responses to inflammatory stimuli and aberrantly expressed miRNAs regulating phenotypic switch of macrophages [84]. They demonstrated that miR-125a-3p and miR-26a-2 promote M1-like phenotype; miR-193b facilitates M2a polarization; or miR-29b-1, miR-27a, miR-222, and miR-132 enforce polarization towards M2b-like phenotype; respectively [84]. TAMs mostly belonging to the M2d sub-class have been found to be associated with poor prognosis [7,42,113]. Mounting evidence from recent studies has shown that many signaling molecules such as phosphatases, cytokines, kinases, receptors, and miRNAs regulated by various signaling mechanisms may control the macrophage phenotypic switch [115,116]. This activation and polarization of macrophages has been dictated by TME signals which recruit the transcription factors. It has been reported that STAT-1, IRF-5, NF-κB pathway, AKT2, and their downstream target genes such as TNF-α, TLRs, IL-1, IL-6, IL-12, CCL2, and CXCL10 are critical for maintaining M1-like phenotype while STAT-6, IL-4R, IRF-4, peroxisome proliferator-activated receptor δ (PPARδ), PPARγ, and Jumonji domain-containing protein 3 (JMJD3) promote the M2-like phenotype [115]. These macrophage polarizations are modulated by several miRNAs, which further provide potential targets to manipulate the macrophage function. Figure 1 and Table 1 explain the miRNAs regulating macrophage activation and polarization.

Based on the molecular signals received from TME, macrophages exhibit a continuum of phenotypes to perform different cellular and biochemical functions. We can have a general classification of macrophages by grouping them into M1 and M2-like phenotypes. Furthermore, on the basis of activation status and functional heterogeneity, M2-like macrophages may be categorized into four subsets: M2a, M2b, M2c, and M2d [42]. The detailed description of these subsets has been given in Table 2. TAMs constitute the major portion as inflammatory cells in TME; they display the similar morphological and functional characteristics as M2-like macrophages [5,7,113]. Therefore, TAMs are recognized as new subgroup of M2d [42]. Of note, the clear classification of macrophages subtypes is complex. It is difficult to identify a specific type of macrophages using single set of specific markers. One main feature of all M2-like macrophages is the generation of ARG-1 enzyme that reduces T-cell activation [55,114]. Graff and co-workers performed the miRNA profiling studies on mammalian macrophages to identify the miRNAs associated with macrophage responses to inflammatory stimuli and aberrantly expressed miRNAs regulating phenotypic switch of macrophages [84]. They demonstrated that miR-125a-3p and miR-26a-2 promote M1-like phenotype; miR-193b facilitates M2a polarization; or miR-29b-1, miR-27a, miR-222, and miR-132 enforce polarization towards M2b-like phenotype; respectively [84]. TAMs mostly belonging to the M2d sub-class have been found to be associated with poor prognosis [7,42,113]. Mounting evidence from recent studies has shown that many signaling molecules such as phosphatases, cytokines, kinases, receptors, and miRNAs regulated by various signaling mechanisms may control the macrophage phenotypic switch [115,116]. This activation and polarization of macrophages has been dictated by TME signals which recruit the transcription factors. It has been reported that STAT-1, IRF-5, NF-κB pathway, AKT2, and their downstream target genes such as TNF-α, TLRs, IL-1, IL-6, IL-12, CCL2, and CXCL10 are critical for maintaining M1-like phenotype while STAT-6, IL-4R, IRF-4, peroxisome proliferator-activated receptor δ (PPARδ), PPARγ, and Jumonji domain-containing protein 3 (JMJD3) promote the M2-like phenotype [115]. These macrophage polarizations are modulated by several miRNAs, which further provide potential targets to manipulate the macrophage function. Figure 1 and Table 1 explain the miRNAs regulating macrophage activation and polarization.

A plethora of cytokines and chemokines expressed in TME, such as TNF-α, TNF-β, CCL2, CSF-1, IL-4, IL-10, VEGF, and angiopoietin-2 (ANG-2) perform an indispensable function in macrophage differentiation, maturation, plasticity, and activation [13,36]. Interestingly, miRNAs regulate various transcription factors that control macrophage plasticity and activation. To understand the significance of miRNAs in the activation and polarization of macrophages, Zhang et al. used M1 and M2-like macrophages isolated from mouse bone marrow to determine the miRNAs associated with macrophage phenotypic switch. By performing miRNA profiling, they demonstrated that M1-like phenotype was accompanied by enhanced intracellular levels of miRNAs, such as miR-204-5p, miR-451, miR-155-5p, and miR-181a, and reduced expression of miR-143-3p, miR-125-5p, miR-146a-3p, and miR-145 [125]. In this study miRNA signatures associated with differentiation and polarization of mouse bone marrow-derived macrophages were established, which may be used as potential targets for re-programming of macrophages. In another study, Jiménez et al. performed miRNA profiling to unravel the miRNAs associated with macrophages differentiation and polarization in human monocytes. They demonstrated four highly expressed miRNAs: miR-181a- 5p, miR-125b-5p, miR-125a-5p, and miR-193b-3p associated with M1-like phenotype [70]. In the same study, elevated expression of miR-500a-5p and miR-502-3p, miR-181a-5p and reduced levels of miR-181a were demonstrated in M2a-like macrophages stimulated by IL-4. Whereas in M2c-like macrophages induced by IL-10, enhanced expression of miR-21-5p, miR-146b-5p, and miR-22-3p and diminished expression of miR-200a-3p and miR-339-3p were noticed. Apart from these studies, several miRNAs have been identified which can participate in macrophages development process. For instance, overexpression of miR-124 in macrophages derived from mouse bone marrow promotes M2-like phenotype as evident by higher expression of markers such as ARG-1 and FIZZI-1 associated with M2-like phenotype [126]. Similarly, overexpression of miR-146a in peritoneal macrophages promotes the alternative pathway via targeting PPARγ, inhibin βA subunit of activin A (INHBA), and Notch-1 [127,128]. MiRNAs facilitate macrophage phenotype switching via targeting various transcription factors. It has been demonstrated that miR-127 and miR-125b promote the M1-like phenotype via targeting Bcl-6 and IRF-4, respectively [129,130]. Some other miRNAs impacting macrophage polarization via regulating expression of transcription factors/activators have been shown in Figure 1. Depending on the stimuli received from TME, the impact of few miRNAs such as miR-142, miR-125a/b, and miR-155 on macrophage polarization (M1 to M2 transition) might be bidirectional. The detailed mechanism of these miRNAs has been reviewed by [88,131]. Since different sets of miRNAs regulate TAM phenotypic transition; this offers a unique opportunity to manipulate the functions of TAMs. For instance, enforced expression of miRNAs that promote re-education of macrophages from M2 to M1-like phenotype could be helpful to suppress cancer cell growth and division.

Moreover, the re-education of TAMs might also activate other tumor cell killing cells. It will also be interesting to develop small molecules/therapeutics that could regulate the activity of a specific set of miRNAs to manipulate macrophage functions. Macrophage polarization regulation is a very complicated process that involves cooperative action of multiple factors such as cytokines, growth factors, chemokines, transcription factors, et cetera. The regulatory roles of miRNAs on these factors add more complexity. Because of the involvement of integrated miRNAs networks in TAM polarization and activation, it would be interesting to unravel how multiple miRNAs act in a cooperative manner under in vivo conditions.

## 5. MiRNAs Involved in Regulation of Recruitment, Infiltration and Immunosuppressive Function of Macrophages

It has been shown that a subset of the immune cells like MDSCs, macrophages, and DCs are attracted by active secretion of a series of chemokines by tumor cells. Notably, circulating monocytes are recruited by CCL2-CCR2 signaling. Apart from that, CCL5-CCR5 signaling axis in macrophages is associated with advanced stages of different tumor types. Under the hypoxic condition, CXCL12 secreted by tumor cells causes infiltration of CXCR4 expressing TAMs in solid tumors. Some other chemokines such as CCL7, CX3CL1, and factors like M-CSF and VEGF are also involved in TAMs migration and recruitment [132,133]. MiRNAs can also act as a determinant in macrophage recruitment at tumor site via producing various growth factors, chemokines, cytokines, et cetera. [4,88]. Chen et al. reviewed the critical role of miRNAs in the recruitment of monocytes/macrophages at the tumor location [88]. It is suggested that TAMs constitute up to 50% of total TME [134,135,136]. Enhanced levels of miR-375 were noticed in breast cancer cells, which promote the recruitment of macrophages via modulating CCL2. Frank et al. demonstrated the influence of miR-375 expression on recruitment and infiltration of macrophages using tumor spheroid and in vivo xenograft models [71].

Moreover, the authors concluded that during apoptosis, breast cancer cells transfer miR-375 to TAMs. This process increases the infiltration and recruitment of macrophages into the TME [71]. Thus, miR-375 can modulate the tumor development process in breast cancer [71]. Contrary to this, an inverse correlation has been demonstrated between miR-125b and TAMs in testicular germ cell tumors (TGCTs). Overexpression of miR-125b in testicular tumor cells significantly reduced the TAMs population at tumor site via inhibiting the production of tumor-promoting cytokines such as CSF-1 and CX3CL1 [112]. In HCC, a low level of miR-148b enhances CSF-1 expression, which leads to TAMs infiltration and metastasis [111]. Another study performed by Chai et al. on HCC has shown that miR-26a inhibits recruitment of macrophages at tumor sites via targeting M-CSF [137]. The present study also suggests that the expression level of miR-26a might be used as a prognostic biomarker for the determination of cancer grade.

TAMs are mostly known to play immunosuppressive roles in TME [45]. MiRNAs, as multifaceted modulators affect the function of cells present in TME including TAMs via influencing the levels of immune modulators. Knockdown of miR-155 in TAMs enhances the IL-10 production, which increases their immunosuppressive abilities [138].

## 6. Extracellular MiRNAs Secreted from TAMs

**Figure 2:**
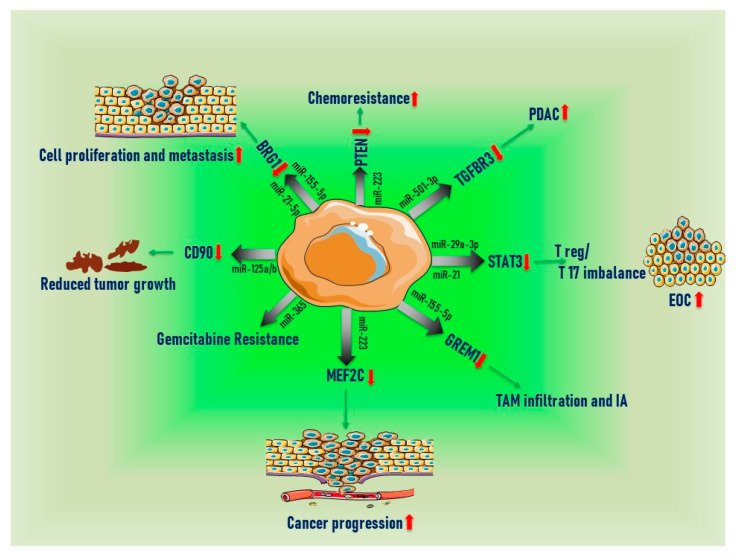
Extracellular miRNAs secreted from TAMs. (red arrows showing the increase or decrease in target gene expression or tumor growth).

Tumor cells in the TME usually secrete a lot of soluble extracellular vesicles (EVs), like microvesicles and exosomes. Exosomes and microvesicles are composed of lipids, proteins, ncRNAs and nucleic acids. They are well-known to play a significant role in cell to cell communication [139,140]. In general, microvesicles are larger (>100 nm) in diameter as compared to exosomes, and generated from plasma membrane, whereas exosomes consist of lipid bilayer membrane, are smaller in size (below 50–100 nm), and derived from late endosomes or multi-vesicular bodies (MVBs). Exosomes are a significant mediator of intercellular communications for both pathological and physiological conditions; therefore, tumor cell-derived exosomes always demanded the attention of cancer biology researchers. From research within the last few years, exosomes derived miRNAs were shown to mediate cellular communications within the TME and can carry out immune modulation to a different extent [141]. In epithelial ovarian cancer (EOC), TAMs derived exosomes loaded with miR-29a-3p and miR21-5p directly inhibit STAT3 signaling in CD4+T cells. Inhibition of STAT3 signaling reduces IL-4, IL-6, and TNF-α production with a significant increase in anti-inflammatory IL-10 levels, creating an imbalance between Treg/Th17 cells, thus establishing the immunosuppressive microenvironment which promotes ovarian cancer progression [104]. Based on these results, it was demonstrated that targeting these miRNAs or exosomes might be an effective approach to inhibit ovarian cancer progression. Lan et al. showed that in colon cancer, TAMs derived exosomes enriched with miR 21-5p and miR155-5p increase the tumor cell migration and invasion [105]. MiR-21-5p also creates an immunosuppressive environment in epithelial ovarian carcinoma [104]. In HCC, exosomal miR125a/b released from TAMs in the TME negatively influences tumor cell division and stemness properties by targeting CD90 [106]. Wang et al. suggested that exosomes containing miR-125a/b could be potential therapeutic targets to design next-generation therapeutics for HCC patients [106]. Whereas, exosomal-miR-21 secreted from M2-like macrophages imparts chemoresistance in gastric cancer cells by modulating the PI3K/Akt axis [107]. In the same study, it was shown that targeting miR-21 derived from TAMs could be a potential approach to suppress chemoresistance in gastric cancer patients. Similarly, in EOC, miR-7 carrying exosomes derived from TAMs inhibit metastasis by targeting the EFGR/AKT pathway [102]. In another study, Feng et al. observed that miR-155-5p loaded exosomes released from TAMs promote intracranial aneurysm (IA) formation and TAM infiltration via targeting Gremlin 1 (GREM1) [142]. Furthermore, the tumor-promoting role of exosomes enriched with miR-155-5p and miR-21-5p was noticed in colorectal cancer. This report has shown miR-155-5p and miR-21-5p released from M2-like macrophages enhances tumor cell proliferation and metastasis [105]. Uptake of exosomes containing miR-365 by PDAC cells confers resistance to gemcitabine [103]. This study highlights the importance of exosomes for therapeutic purposes. Exploiting the exosomes for delivering the therapeutic agents, including miRNAs antagonists represents the new approach for cancer therapy. In addition, the tumor-promoting role of miR-223 via modulating myocyte-specific enhancer factor 2C (MEF2C) has been established in breast cancer cells [143]. Furthermore, another research group has shown that exosomes loaded with miR-223 can induce the chemoresistant phenotype in EOC cells [109]. Based on the results, the authors demonstrated that the exosomes loaded with miR-223 might be used as a prognostic biomarker for the progression of ovarian carcinoma. Exosomal miR-365 released from TAMs in pancreatic adenocarcinoma induces gemcitabine chemoresistance by activating the cytidine deaminase [103]. A schematic diagram showing the release of extracellular miRNAs from M2-like macrophages has been given in Figure 2. With all these unusual complex interventions of miRNAs loaded in exosomes derived from TAMs in cancer progression, more and more research is required to characterize miRNAs from TAMs and other competent immune cells.

## 7. Extracellular miRNAs Secreted from Cancer Cells

**Figure 3:**
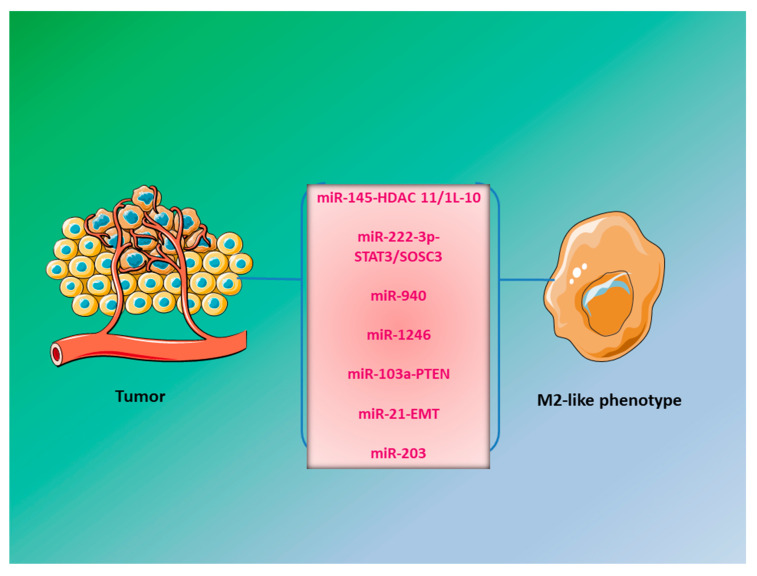
miRNAs secreted from cancer cells promoting the M2-like macrophages.

Innate immune cells always play a considerable role in the regulation of multistep cancer development. Short ncRNAs such as miRNAs have continued to be in the spotlight for the last three decades as they are the critical modulators of transcription and translation processes within the cell [144]. MiRNAs secreted from tumor cells affect the cells present in TME including TAMs, thus regulate the metastasis process of the tumor. An important role of exosomal/EVs- miRNAs released from cancer cells is to regulate macrophage phenotypic switch. Several exosomal extracellular miRNAs have been reported to regulate this process, for instance, exosomes loaded with miR-222-3p derived from EOC cells induce the M2-like phenotype via regulating the SOCS3/STAT3 pathway [76]. In another study, exosomes containing miR-940 released from ovarian cancer cells under hypoxic conditions induce M2-like phenotype in macrophages, which increases ovarian cancer progression [77]. Colon cancer cells exhibiting the gain of function p53 mutation secrete exosomes enriched in miR-1246, which promote M2 polarization of macrophages, thus creating a tumor supportive environment [145]. In CRC, Takano et al. have shown that exosomes enriched in miR-203 could help in the differentiation of monocytes to TAMs, which may impart enhanced cell growth, proliferation, and migration. Additionally, exosomal miRNA-203 in serum could be used as a prognostic biomarker for cancer progression and metastasis, and suppression of miR-203 might be a potential approach to combat CRC progression [78]. Macrophages are also polarized by exosomes carrying miR-let7a released from hypoxic cancer cells. MiR-let7a induces M2-like phenotype in bone-marrow-derived macrophages via targeting the insulin/AKT/m-TOR signaling pathway, which enhances the tumor progression [85]. Activation of transcription factor Snail, that regulates EMT process, increases the production of exosomal miR-21. Uptake of exosomes loaded with miR-21 by CD14+ human monocytes suppresses the M1-like macrophages and induces a switch to the M2-like phenotype [146]. Hsu et al. demonstrated an enrichment in M2-like macrophages which was accompanied by transfer of miR-103a loaded EVs from hypoxic lung cancer cells to macrophages. They observed that miR-103a downregulates tumor suppressor gene (PTEN) in macrophages which in turn activated AKT/STAT3 pathways as well as multiple angiogenic, immunosuppressive, and tumor-promoting factors [81]. Under the hypoxic conditions, cancer-cell derived miR-125b-5p, miR-21-2p, and miR-181d-5p promote M2-like phenotype of macrophages, which may be involved in cancer initiation, angiogenesis, and metastasis [82]. A shift to the M2-like phenotype has been observed in macrophages when they uptake exosomes with elevated expression of miR-503 from glioma cells [147]. A few miRNAs secreted from cancer cells promoting M2-like phenotype have been shown in Figure 3.

It has been shown that miR-19a-3p secreted by breast cancer cells increases VEGF expression and STAT3, inducing M2 to M1 polarization of TAMs [148]. Similarly, glioma cell-derived EVs containing miR-21 and miR-451 induce the shifting of the cytokine profile of macrophage towards immunosuppressive phenotype [92]. In glioblastoma, extracellular miRNAs serve as promising prognostic and diagnostic markers in cancer biology and can unravel a more robust approach to defeat this disease. Few miRNAs, for example, miR-29a and miR-21 derived from lung tumor cells, can promote tumor progression via activating the Toll-like receptor (TLR) mediated inflammatory response [101]. Furthermore, Challagundla et al. have shown a positive association between two tumor-promoting miRNAs; miR-155 and miR-21. Concerted action of these two miRNAs contributes to chemoresistance of cancer cells [95]. In addition, uptake of EVs enriched with miR-145 derived from colorectal cancer cells by macrophages increases their oncogenic effects via downregulation of histone deacetylase 11 (HDAC11) [79]. Guo et al. found that exosomal transfer of miR-20a-5p released from breast cancer cells facilitate breast cancer development and metastasis [149]. They demonstrated that exosomal transfer of miR-20a-5p promotes migration of breast tumor cells to bone [149]. In another study performed on breast cancer cells depicts that transfer of extracellular miR-375 from tumor cells to TAMs induced their migration and infiltration [71]. In cancer cells, it has been shown that the treatment of anti-cancerous compound epigallocatechin gallate (EGCG), enhanced the level of miR-16 in both tumor cells as well as exosomes derived from cancer cells. Exosomal miR-16 was engulfed by M2-like macrophages, which induced M2 to M1 polarization of macrophages via inhibiting the NF-kB pathway [80]. In addition treatment of EGCG reduced the migration and infiltration of M2-like macrophages via curtailing the expression of certain immunomodulators [80]. In pancreatic cancer, extracellular miRNAs such as miR-155, miR-125b-2 promote M1-like phenotype while under oxygen deprived condition, miR-301a-3p triggered M2-like phenotype via modulating PTEN/PI3Kγ axis [74,83]. In HCC, miR-23a-3p from liver cancer cells activates Akt, which increases inflammatory cytokines and PD-L1 expression [96]. All these studies suggest that tumor cells secrete extracellular miRNAs to communicate with TAMs and other cells present in TME. These miRNAs regulate key signaling molecules in TAMs and ultimately decide the phenotype and function of TAMs. Manipulation of macrophages such as M2 to M1 polarization may be a potential approach to combat cancer.

## 8. Conclusions and Future Perspective

The signaling events and biology of TME are well understood from previous studies, however, little is known about how TAMs and other cells present in TME create a niche for cancer progression. Recently, an emerging shred of evidence suggests the implication of miRNAs in this process. In the last decade, a series of studies have shed light on the underlying mechanisms driven by miRNA to elicit early phases of carcinogenesis and inflammatory response. Unraveling the link between miRNA and cancer progression process may open the door to inventing new diagnostic and therapeutic strategies. This article focuses on the significance of miRNAs in macrophage differentiation, maturation, and functions. MiRNAs regulate almost all the biological and physiological processes associated with macrophages. Most of the studies related to these processes are based on the mechanistic investigation of individual miRNAs. However, macrophage development and functions are facilitated by the cooperative action of multiple miRNAs. It would be fascinating to study how various miRNAs cooperate to control cellular and molecular signaling inside the macrophages. Since individual miRNAs may have multiple target genes, thus it could regulate several biological processes. Hence, understanding the integrated networks modulated by the concerted action of numerous miRNAs represents a challenge. Inevitable outcomes in this field may provide a new paradigm regulating cancer progression and metastasis and eventually uncover new pharmacological targets to inhibit carcinogenesis. Many microarray analysis have been done to understand the signaling networks inside the tumor cells, however, a series of studies have confirmed the connection between altered expression of miRNAs inside the tumor-infiltrating cells, including macrophages and cancer initiation and progression. Further identification of new miRNAs and their functional characterization might uncover new avenues to design miRNA-based therapeutics. Altered expression of miRNAs may regulate TAMs function, thus could impact the cancer development process. Since TAMs have lower mutation rates and heterogeneity than cancer cells, depletion or re-polarization of TAMs via modulating the expression of a specific set of miRNAs present a beneficial approach in cancer therapy. Thus, restoring the normal levels of miRNAs could be a possible strategy to improve patient outcomes. Delivering miRNAs mimics or antagonists by nanoparticles or antibody-linked nanoparticles bear significant potential as a therapeutic strategy.

## Figures and Tables

**Table 1:** List of miRNAs regulating macrophage function.

miRNAs Involved in Macrophage Polarization	Function	Reference
miR-375	Facilitates macrophage recruitment, M2-like phenotype and tumor progression	[71]
miR-23a/miR-27a/miR24-2 cluster	Promote M1-like phenotype, inhibit M2-like phenotype in breast cancer	[72]
miR-340-5p	Promotes M2 polarization in glioblastoma	[73]
miR-155, miR-125b-2	Macrophage re-programming to M1-like phenotype in pancreatic cancer	[74]
miR-29a-3p	Promotes M2 polarization in OSCC	[75]
miR-222-3p	Promotes M2-like phenotype in ovarian cancer	[76]
miR-940	Promotes M2 polarization in epithelial ovarian cell carcinoma	[77]
miR-203	Promotes M2 polarization and metastasis in CRC	[78]
miR-145	Promotes M2-like phenotype in CRC	[79]
miR-16	Promotes M1-like phenotype in breast cancer	[80]
miR-103a	Increases M2 polarization in lung cancer	[81]
miR-21-3p, miR-181d-5p, miR-125b -5p	Promote M2 polarization, cancer cell migration, proliferation in EOC	[82]
miR-301a-3p	Enriches M2-like macrophages via modulating PTEN/PI3Kγ axis in pancreatic cancer	[83]
miR-132, miR-29b-1, miR-27a, miR-146a, miR-222	Higher expression of these miRNAs promote M2b-like phenotype	[59,84]
miR-let7a, miR-320a, miR-146a	Promote M2-like phenotype	[85,86,87]
miR-142-3p	Inhibit M2 polarization, reduces tumor growth in HCC	[88,89]
let-7c	Inhibits M1 polarization and promote M2-like phenotype	[90]
miR-1246	Promote M2 polarization via modulating STAT3 and NF-κB axis.	[88,91]
let-7d-5p	Promote M2-like phenotype	[16]
miR-451, miR-21	Influence macrophage polarization in glioblastoma	[92]
**Extracellular miRNAs Secreted from Cancer Cells Regulating Cancer Progression or Chemoresistance**	**Function**	**Reference**
miR-221, miR-222	Promote tamoxifen resistance in breast tumor cells	[93]
miR-1246	Imparts chemoresistance in ovarian cancer cells	[94]
miR-21	Induces chemoresistance in neuroblastoma, enhances miR-155 expression	[95]
miR-23a-3p	Promotes tumor cell escape via impairing T-cell function	[96]
miR-204-5p	Regulates cisplatin resistance in EOC	[97]
miR-let7a	Creates an immunosuppressive environment by enhancing the expression of M2-like phenotype associated genes	[85]
miR-142-3p	Induces malignant phenotype in oral carcinoma	[98]
miR-105	Promotes tumor progression and metastasis via degrading vascular endothelial barriers	[99]
miR-214	Promotes tumor growth via deregulating PTEN and impairing T-cell function in mouse model	[100]
miR-21, miR-29a	Induces inflammatory response in NSCLC cells via activating NF-kB pathway	[101]
**Extracellular miRNAs Derived from TAMs Regulating Cancer Progression or Chemoresistance**	**Function**	**Reference**
miR-7	Inhibits metastasis in EOC via modulating EGFR/AKT/ERK1/2 axis	[102]
miR-365	Promotes gemcitabine chemoresistance in PDAC	[103]
miR-29a-3p, miR-21-5p	Establish an immunosuppressive environment in EOC	[104]
miR-155-5p, miR-21-5p	Increase migration and invasion of colon cancer cells via downregulating BRG 1	[105]
miR-125a/b	Negatively influence tumor cell division and stemness properties of HCC via targeting CD90	[106]
miR-21	Induces chemoresistance in gastric cancer	[107]
miR-501-3p	Progression of PDAC via modulating TGF-β	[108]
miR-223	Imparts cisplatin resistance in EOC	[109]
**miRNAs Involved in Macrophage Differentiation and Maturation**	**Function**	**Reference**
miR-155, miR-146a, miR-338, and miR-342	Facilitate the progression of HSCs differentiation process	[66]
miR-17-92 cluster: miR-18a, miR-17, miR-92a, miR-19a, miR-19b-1, miR-20a	Inhibition of their expression by PU.1 promote HSCs differentiation	[67]
miR-146a, miR-126, miR-29a, miR-155, miR-130a, miR-125a/b, miR-338, miR-342, miR-21, miR-196b	Mediate differentiation and maturation of HSCs by regulating expression of various target genes	[61,63,64,66,69,110]
**miRNAs Regulating Recruitment of Macrophages at Tumor Site**	**Function**	**Reference**
miR148b	Inhibits TAM infiltration in tumor	[111]
miR-375	Induces TAM infiltration in breast cancer	[71]
miR-125b	Reduces recruitment of macrophages at tumor site	[112]

**Table 2:** Classification of M2-like macrophages.

Subtype	Functions	Key Activating Stimuli	Markers	References
M2a	Anti-inflammatory and tissue repair, killing of the infectious parasites	M-CSF, IL-13, IL-14	CD206, MHC-II, FZZI, CD163, Arg-1, IL-10, TGF-β, WNT5b	[117,118,119,120,121,122,123,124]
M2b	Increases infection, immunoregulation, tumor growth and progression	TLR, IL-1R antagonist, immunocomplexes	CD206, CD86, IL-6, IL-1, IL-10 TNF-α	
M2c	Immunosuppression, phagocytosis, tissue remodeling, matrix deposition, and efferocytosis	IL-10, glucocorticoids	CD206, CD163, IL-10, MERTK, ECM, TGF-β	
M2d	Angiogenesis, anti and pro-tumoral properties	A2AR ligands, TLR, IL-6	IL-10, IL-12, VEGF, TGF-β	

## Abbreviations

A2AR	Adenosine A2A receptor
ANG2	Angiopoietin-2
BAK1	BCL2 antagonist/Killer 1
CCL2	C-C motif chemokine ligand 2
CRC	Colorectal cancer
CSF-1	Colony-stimulating factor-1
EGCG	Epigallocatechin gallate
EGF	Epidermal growth Factor
Egr2	Early growth response protein 2
EMT	Epithelial to mesenchymal transition
EOC	Epithelial ovarian cancer
EVs	Extracellular vesicles
GATA-3	GATA binding protein-3
GMP	Granulocyte–monocyte progenitor
GSK3β	Glycogen synthase kinase 3 beta
HCC	Hepatocellular carcinoma
HIF-2α	Hypoxia-inducible factor-2 alpha
ILs	Interleukins
MDSCs	Myeloid-derived suppressor cells
MERTK	MER proto-oncogene, tyrosine kinase
NF-kB	Nuclear factor kappa-light-chain-enhancer of activated B cells
NSCLC	Non-small-cell lung carcinoma
OSCC	Oral squamous cells carcinoma
PDAC	Pancreatic ductal adenocarcinoma
PD-L-1	Programmed death-ligand-1
PI3K	Phosphatidylinositol 3-kinase
PPARδ	Peroxisome proliferator-activated receptor δ
SOCS3	Suppressor of cytokine signaling 3
STAT-3	Signal transducer and activator of transcription-3
TGCTs	Testicular germ cell tumors
TGFBR3	Transforming Growth Factor Beta Receptor 3
TLR	Toll like receptor
TNF-α	Tumor-necrosis factor-α
TWIST1	Twist-related protein 1
uPA	Urokinase-type plasminogen activator
VEGF	Vascular endothelial growth factor
WNT5B	Wnt family member 5B
